# Infant sleeping arrangements and cultural values among contemporary Japanese mothers

**DOI:** 10.3389/fpsyg.2014.00718

**Published:** 2014-08-19

**Authors:** Mina Shimizu, Heejung Park, Patricia M. Greenfield

**Affiliations:** ^1^Department of Human Development and Family Studies, The Pennsylvania State University, University ParkPA, USA; ^2^Department of Psychology, University of CaliforniaLos Angeles, CA, USA

**Keywords:** social change, cultural values, sleeping arrangements, parenting practices, parental ethnotheory, socialization, gender roles, Japan

## Abstract

We examined infant sleeping arrangements and cultural values of Japanese mothers in 2008 and 2009. Based on Greenfield's theory of social change and human development, we predicted that social change in Japan over the last decades (higher economic and education level, urbanization, complex technology, more women in the work force) would lead to a decline in mother-infant co-sleeping, compared with published findings concerning Japanese sleeping arrangements in the 1960s and 1980s. We also predicted that the practice of having babies sleep in their own beds and/or own rooms would be supported by ethnotheories stressing infant independence and other values adaptive in an urban, technologically sophisticated, relatively wealthy, and highly educated populace. Fifty-one Japanese mothers' comments posted on Internet parenting forums were analyzed. Contrary to our hypothesis, co-sleeping was as frequent among Japanese mothers in 2008-2009 as it had been in the 1960s and 1980s. However, analysis of the values of co-sleeping mothers revealed frequent discrepancies between values and practices. In contrast, the minority of mothers whose babies slept alone in a separate room all expressed consonant values. Our qualitative analysis indicates that it is not always easy for Japanese mothers to construct values for child rearing and gender roles that integrate traditional infant care practices with current sociodemographic conditions.

## Introduction

Japan is thought to emphasize collectivism, interdependence, and solidarity, whereas the United States (US) is thought to value individualism, independence, and autonomy (Hofstede, 1980; Markus and Kitayama, 1991). This dichotomy traces its origin to the 1960s, when Caudill and Plath (1966) argued that infant sleeping arrangements in Japan (parents and infants sleeping together) reflected interdependent/collectivistic values, whereas sleeping arrangements in the US (babies sleeping alone) reflected independent/individualistic values. Research in other countries such as Guatemala has supported this point (e.g., Morelli et al., 1992).

However, recent research and theory suggest that cultural values and children's learning environments involve adaptations to particular sociodemographic conditions (Keller, 2007). When these conditions change, cultural values, and socialization practices change accordingly (Kağitçbaşl, 2005; Greenfield, 2009), modifying the ecological niche of the developing child (Super and Harkness, 1986). Cultural values affect the developmental niche through parental ethnotheories: folk theories of good parenting and ideal children. They are the nexus through which elements of the larger culture are filtered (Harkness et al., 2001). Globally, environments have shifted from rural to urban, from subsistence to commerce, from poorer to wealthier, from simple to complex technology, from homogeneity to ethnic diversity, and from informal education at home to formal schooling (Greenfield, 2009). At the extremes, the former kinds of social environment can be summarized as *Gemeinschaft* (community), while the latter can be summarized as *Gesellschaft* (society) (Tönnies, 1887/1957).

Greenfield (2009) presents a multilevel theory of social change and human development. Sociodemographic conditions are at the top level; at the next two levels down, values and socializing practices adapt to sociodemographic conditions. So that their children's behavior will be adaptive to a *Gemeinschaft* environment, parents living in a *Gemeinschaft* world socialize their children for interdependence, gender role hierarchy, and complementary gender roles ascribed by birth. So that their children's behavior will be adaptive to a *Gesellschaft* environment, parents living in a *Gesellschaft* world socialize independence; gender ideals are more egalitarian; and gender roles are ideally chosen rather than ascribed by birth (Greenfield, 2009; Manago et al., 2014). Likewise, perspectives are more diverse and technological intelligence more important as adaptations to *Gesellschaft* conditions (Greenfield, 2009; Manago and Greenfield, 2011). When sociodemographic conditions shift, so do the corresponding values and socializing practices. For example, when society shifts in a *Gesellschaft* direction, children become more independent, while gender roles tend to become more chosen and egalitarian (Greenfield, 2009; Manago et al., 2014).

At home, which is “the most fertile ground for the transmission, maintenance, and renewal of culture” (Li, 2012, p. 223), development toward an interdependent or independent pathway starts early on with infant caregiving practices (Greenfield et al., 2003), one of which is infant sleeping arrangements. For instance, mothers valuing interdependence anticipate babies' needs through continuous nursing and staying in physical proximity with babies throughout the night (Brazelton et al., 1969). Such infant caregiving practices provide continuous hydration or the warmth of another human being for temperature regulation, in line with the notion of *Gemeinschaft* environment adaptations. On the other hand, the infant caregiving practice of letting babies sleep alone aligns with independent socialization as a means of adaptation to *Gesellschaft* conditions. For example, mothers respond to babies when they express their needs through crying rather than constantly monitoring babies' needs (Keller, 2007), which allows more sleep for working mothers who require uninterrupted sleep. Co-sleeping does happen in *Gesellschaft* environments, but it is often a last resort for parents occurring when initiated or strongly desired by the child or when the child is ill (Shweder et al., 1995; Welles-Nystrom, 2005). Even with the development of philosophies that encourage bedsharing in the US, co-sleeping in middle-class US environments was much less frequent than in *Gemeinschaft* environments, such as an African village (Weisner et al., 1983). Moreover, it is important to note that *Gesellschaft* environments are complex societies that can contain pockets of more *Gemeinschaft* communities, such as rural towns within the developed US. In rural Kentucky, 71% of children between 2 months and 2 years slept with a parent (Abbott, 1992), a higher rate than in the general US population of the same era (McKenna and McDade, 2005).

## Social change in japan and the research questions

Since World War II, Japan has transformed from a relatively poor, predominantly rural, agricultural country with simple technology, low levels of formal education, and extended family structures (relatively *Gemeinschaft*) into a wealthy urbanized country with advanced technology, high levels of formal education, and a nuclear family structure (relatively *Gesellschaft*) (Ministry of Education, Culture, Sports, Science, and Technology, 2009; World Bank, 2010). Women's roles have been particularly altered, with many more women entering the workforce (Yashiro, 2001). Thus, we asked: (1) Would parenting values and gender roles in Japan move away from roles ascribed by birth (e.g., females take care of children; males work outside the home, Rice, 2001) toward roles based on choice and individual differences? (2) Would parental ethnotheories switch from favoring infant-parent co-sleeping to favoring independent infant sleeping? (3) Would infant sleeping arrangements concomitantly shift from co-sleeping to sleeping apart? Most importantly, we investigated whether Japanese mothers would experience conflict in regards to any of the potential changes explored in questions 1–3.

## Previous research on sleeping arrangements and parental ethnotheories in japan

Despite these sociodemographic changes that dramatically shifted women's roles and family structures, research on Japanese childrearing values heretofore has focused on the interdependence model, ignoring the way in which sociodemographic changes may be shifting cultural values and socialization practices. Focus has remained on intense close relationships between mother and child (i.e., mother-child inseparability) (e.g., Markus and Kitayama, 1991; Lebra, 1994; Rice, 2001). This focus views Japanese mothers as perceiving themselves one with their infants (Greenfield and Suzuki, 1998), thus protecting the baby from stress (Takahashi, 1990). Japanese mothers have been reported as considering sleeping alone merciless in forcing independence on infants (Brazelton, 1990).

Indeed, Japanese children have traditionally slept with parents, especially mothers, in physical proximity (Caudill and Plath, 1966; Caudill and Weinstein, 1969; McKenna and McDade, 2005). In the early 1960s, 90% of the 3–4 month-old infants slept with their mothers in the same room, whereas only 10% slept alone in a separate room from their mothers (Caudill and Plath, 1966). In a subsample of 73 families having two children, 10% of infants slept in their parents' bedding, while 26% of the older children (median age of 3 years) did so. Therefore, most infants and children were in the same room with their parents although in/on a separate bed or *futon* (Japanese sleeping mat).

This proximal sleeping arrangement appears to have been maintained in Japan, even as the society transformed (Ohkubo, 2005). In a cohort married between 1950 and 1954, only 2.6% recalled that their children slept in a separate room; in the cohort married in the early 1990s, none recalled their children sleeping in a separate room. In between the two cohorts, the percentage never went above 4.0%. Nevertheless, given the continuous rapid social change in Japan and around the globe, it is important to investigate the latest 21st century trends. It remains unclear whether the seemingly persistent co-sleeping practice in Japan throughout the 1990s would have finally shifted to show sleeping apart, as Japan has moved even more toward a *Gesellschaft* environment.

## Japanese values in comparative and historical perspective

While parental ethnotheories and gender role values have not been explored in the same studies with infant sleeping arrangements, many studies provide a picture of earlier parenting and gender role ideals in Japan. Fogel et al. (1992) summarized a number of studies of Japanese ethnotheories of infant development that are in harmony with the high rate of infant-parent co-sleeping: “The ideal is for the [Japanese] mother to create a relationship in which the infant is naturally drawn into considerate, interdependent, competent interactions with others. The first step is to satisfy the infant's desires for proximity, to accept and respond directly to the infant's proclivities and affectional needs” (pp. 48–49). These practices constitute early socialization for the Japanese value placed on interpersonal connectedness (Befu, 1986).

Other values, originating in the pre-World War II *Gemeinschaft* environment of Japan, have also been central to traditional ethnotheories of parenting in Japan. For instance, commitment to one's assigned role is an important Japanese value (Lebra, 1976; Rice, 2001), and commitment to the maternal role has had the highest priority for women (Befu, 1986). However, with continuous shift toward *Gesellschaft* conditions, assumptions cannot be made that these values concerning parenting are maintained in the 21st century Japan.

Indeed, this value of interdependence in Japan contrasts with the focus on independence from infancy in countries with a more *Gesellschaft* history such as the US and Germany (Azuma et al., 1981; Keller, 2007). In harmony with these values is the use of objects to provide security, for example, transitional objects like stuffed animals to help the baby fall asleep (Morelli et al., 1992). The maternal role (whether or not to become a mother) is considered a matter of choice, allowing for individual differences (DePaulo, 2006). Egalitarian roles for men and women vis-à-vis childcare also develop (Pruett, 1987).

Indeed, as Japan has moved toward more Gesellschaft conditions, gender roles have changed in this same direction. Rosenberger (2001) has observed that college-educated Japanese women postpone marriage, thereby creating a new category of unmarried womanhood, a category that translates to erosion of traditional interdependent, family-centered values (Manago et al., 2014). In the new generation, women's roles are more by choice than they are ascribed by birth as wife and mother; women's personal achievement often replaces social responsibility as a life-course value (Efron, 2001; Hirao, 2001; Greenfield, 2009). Our inferences in the present study about changes in parenting ethnotheories and gender-role values will be based on these earlier studies depicting both traditional and changing values in Japan.

Ours is the first research on infant sleeping practices in Japan to study both ethnotheories and practices in the same sample. Sleeping practices refer to the conditions under which children are put to sleep. For our purposes the major distinction is whether children sleep alone in their room or in the same room with one or both parents. Earlier researchers drew conclusions about parental ethnotheories based exclusively on sleeping practices (Caudill and Plath, 1966; Caudill and Weinstein, 1969; Wolf et al., 1996; Ohkubo, 2005). Yet, in times of rapid social change, one cannot take for granted complete harmony between values and practices (Greenfield, 2009). Therefore, the relationship between the two is central to how social change is negotiated by individual mothers. To explore this relationship is a major goal of the present study.

## The present study

Given the continuing sociodemographic change in Japan in the *Gesellschaft* direction, we predicted a decrease in co-sleeping among contemporary Japanese mothers, comparing current practices with data from studies of the 1960s and 1980s. More importantly, we examined parental ethnotheories relating to infant sleeping arrangements and associated issues of child development, parenting, and gender roles. We hypothesized that mothers in contemporary Japan would express *Gesellschaft*-adapted ethnotheories more than *Gemeinschaft*-adapted ethnotheories. We implemented a mixed-method approach that allowed us to (1) quantitatively describe sleeping arrangement patterns and maternal values, and (2) qualitatively investigate the discourse of parental ethnotheories surrounding infant sleep. As such, we were able to also explore the direct association between practice and ethnotheories under the current conditions of social change in Japan.

Our data source was Japanese online parenting forums. The anonymous nature of an online forum provided an important methodological advantage. The Japanese dualism of *honne* (true feeling) and *tatemae* (social expectation) has made it difficult for researchers to elicit Japanese mothers' “true” feelings (Rice, 2001). Because of their anonymity, online parenting forums provided a window into emotions that were sometimes discordant with social expectations. Another methodological strength was the public character of the Internet, which created a cultural environment viewed by an audience—going beyond the behavior of a discrete sample in its significance. Hence, in addition to a study of individual mothers, we considered this also to be a study of cultural environment accessible to other mothers/people in Japan.

## Methods

### Participants

Participants were 51 Japanese mothers who posted comments on Japanese Internet parenting forums and indicated having children up to 2-years-old. To be included in our study, mothers had also to indicate residing in Japan with Japanese husbands. Thirty-seven mothers reported exact ages of their babies (range = 1–24 months, median = 9 months). The remaining 14 mothers indicated having a baby younger than 12 months. At the time of data collection in 2009, Internet penetration in Japan was 75.3% (Internet World Stats, 2009). Therefore, our sample came from a broad base of Japanese Internet users. Given the nature of Internet forums, additional demographic information was not collected or publicly available, such as specific region of residence within Japan. Names used in this article are all pseudonyms.

### Data collection and selection

#### Forum selection criteria

We used a Japanese search engine to identify forums where people posted comments relevant to sleeping arrangements and parenting ethnotheories. Data were collected March 1st, 2009 to April 17th, 2009. The postings analyzed were from 2008 and 2009. Using the keywords, 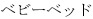
[cribs], 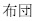 [futons], 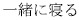 [sleep together], and 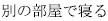 [sleep in a separate room], 39 forums were identified.

The postings analyzed in this study were extracted from threads on the web forums titled pregnancy, childbearing, childrearing, or concerns about childrearing. These electronic bulletin boards were hosted by search engines such as Yahoo and MSN, Japanese newspapers, or knowledge communities, aiming to share, discuss, and potentially solve the concerns of pregnant women and mothers of young children. Any individual could participate in these web forums; they were not targeted to a particular parenting philosophy. Lastly, we eliminated the boards that posted questions biased toward a particular sleeping arrangement (e.g., “I want to ask mothers who sleep with babies on the parental bed.”). Fifteen of the 39 forums met the selection criteria. These 15 forums are listed in the Appendix. The titles of the thread selected from each forum are also included in the Appendix (The Appendix can be found at http://www.frontiersin.org/journal/10.3389/fpsyg.2014.00718/abstract).

#### Comment selection criteria

Within the 15 forums, comments were selected if they included the following words that provided information about sleeping arrangement styles: “futons,” “cribs,” “beds,” “*soine*” (to sleep alongside a person who sleeps) (Niimura, 2008), “separate room,” or “different room.” Comments could not be retrospective recalls of past situations. The resulting comments from 51 mothers were assembled into a database for analysis by the first author in the original Japanese. A subset of comments were translated into English for presentation purposes, as well as for reliability coding (see Reliability coding section below).

#### Description of corpus

The average number of postings per participant was 1.2 (range = 1–7), and the modal response was one posting per participant. The mean number of sentences per posting was 10.31 (range = 3–53). Each of the 15 forums provided only one thread that included one or more of the key words.

### Data analysis

#### Categorization of sleeping arrangement practices

Sleeping practices of Japanese mothers were examined by classifying their discourse about current sleeping arrangements into three categories. The categories were based on physical closeness between a mother and her baby: (1) Within Arms' Reach in the Same Room, (2) Beyond Arms' Reach in the Same Room, and (3) Beyond Arms' Reach in a Separate Room.

Within Arms' Reach in the Same Room included futon or bed-sharing, mother's bedding adjacent to the baby's bedding, and *soine*. The definition of *soine* varied among the participants, such that some defined *soine* as sleeping with a baby on the same bedding, whereas others defined it as sleeping with a baby on adjacent bedding. Yet, both situations share the same defining feature of this category: mother and baby are within arms' reach. This category defined the most proximal sleeping arrangements. It was developed for the Japanese practice of using sleeping mats. The sleeping arrangement that is functionally equivalent to Within Arms' Reach in the Same Room in most other cultures would be bedsharing. Beyond Arms' Reach in a Separate Room (i.e., baby sleeps in a separate room from the mother) included the most distal sleeping arrangements. Beyond Arms' Reach in the Same Room represented an intermediate sleeping distance between mother and baby; this was when baby slept in a crib in the same room as the mother.

#### Interrater reliability for sleeping arrangement practices

To achieve inter-rater reliability, two coders independently inspected 20% of the data (translated into English for the coder who did not know Japanese). They achieved 83% agreement on the selection of sentences that described sleeping arrangement practices. Next, each coder independently categorized sleeping arrangements as (1) Within Arms' Reach in the Same Room, (2) Beyond Arms' Reach in the Same Room, or (3) Beyond Arms' Reach in a Separate Room (kappa = 0.84). This reliability between a coder coding from Japanese and a coder coding the same material from English assured the accuracy of the translations of reports of sleeping arrangement practices.

#### Categorization of parental ethnotheories

Thirty-five mothers (69%) expressed one or more of the eight themes described below. Based on previous research and theory (Keller, 2007; Greenfield, 2009), four pairs of parenting ethnotheory themes were identified. In each pair, the first theme refers to a *Gemeinschaft*-adapted value while the second indicates a *Gesellschaft*-adapted value. Definitions and an example for each theme provide insight into the maternal experience in contemporary Japan. The first two themes are very relevant to sleeping arrangements; the third to childrearing in general, and the fourth to gender roles.

1. Interdependence/Oneness vs. Maternal Independence/Baby as Independent Agent.

Interdependence/Oneness represented unclear boundaries between a mother and a child such that a mother treats her child as extension of self or with adoration, whereas Maternal Independence/Baby as Independent Agent represented a mother's desire for independence from her child or clear boundaries between her and her child.

**Interdependence/Oneness:** *It is a blissful time when I sleep with my baby. It is my great pleasure to see [his/her] adorable face, to hear the breathing, and to touch the soft body*.

**Maternal Independence:** *It is ideal that the baby sleeps in a crib, since I want to sleep well*.

**Baby as Independent Agent:** (From a co-sleeping mother of a three-month-old baby) *Recently I started having [the baby] sleep alone on the futon [without my assistance] around 8:30 pm, I don't go check until [s/he] cries*.

2. Security by Being Close vs. Security Provided by Objects.

Security by Being Close was when a mother indicated that being physically close to her child provided the child's sense of security or sense of security for herself, whereas Security Provided by Objects was when a mother provided objects or technological devices to her child for safety.

**Security by Being Close:** … *on the same futon. [He/she] will sleep well, since [he/she] will feel secure by being close to me*.

**Security Provided by Objects/Technology:** *If you don't feel secure, you can put a camera and microphone in the corner of the crib. Cameras and microphones are well-developed, so you can check baby's breathing from your room*.

3. Adherence to Norms/Maternal Responsibility vs. Individual Differences/Personal Choice.

Adherence to Norms/Maternal Responsibility represented a mother's emphasis on adherence to communally shared opinion, whereas Individual Differences/Personal Choice represented a mother's acknowledgement of individual differences or emphasis on the importance of personal choice.

**Maternal Responsibility:** (In response to a mother whose baby sleeps alone) *To me, it is just irresponsible… If your baby had an accident, who would take responsibility for that?”*

**Individual Differences:** *There are individual differences and family differences. I think, it* [baby's sleeping alone in a separate room] *is acceptable*.

4. Ascribed or Hierarchical Gender Roles vs. Achieved or Egalitarian Gender Roles.

Ascribed or Hierarchical Gender Roles represented a mother's assumption that childrearing is exclusively a part of the maternal role, whereas Achieved or Egalitarian Gender Roles represented a mother's assumption that childrearing involves cooperation between a father and a mother.

**Ascribed gender roles:** *We need my husband to bring in money, so I think of child-rearing as a part of my job*.

**Egalitarian gender roles:** *Child-rearing needs cooperation* [between father and mother]*!*

Coders identified these eight themes in the ethnotheoretic discourse of participants. A given theme was counted only once per participant. For the 35 participants who expressed at least one of the forgoing themes, the mode was one theme per participant; the mean was 1.49 themes per person. Based on the expressed themes, we classified each participant's discourse into one of three cultural models: (1) *Gemeinschaft*-adapted, (2) *Gesellschaft-*adapted, and (3) mixture of *Gemeinschaft*-adapted and *Gesellschaft*-adapted (only possible if more than one theme was expressed). When a participant produced at least one *Gemeinschaft*-adapted and at least one *Gesellschaft*-adapted theme, her cultural model was defined as a mixture. An example of the mixture category is as follows:

**Mixture: Individual Differences (Gesellschaft adaptation) and Security by Being Close (Gemeinschaft adaptation)**. *I think that we don't need to follow what books say, since each family has their own ideas for child-rearing. I think there is no right or wrong. … I do soine with my baby. … I will have him sleep alone in his own room when he starts to express whether he has pain or is hot or cold. He is now four months old, and is not able to roll over yet. … I've seen the futon was removed and sometimes covered over his face. If you are close to your baby, you would be able to notice those situations*.

This participant expressed both a *Gesellschaft*-adapted value (individual differences) and a *Gemeinschaft*-adapted value (security by being close). In contrast to a typical *Gemeinschaft*-adapted value: adherence to communally shared opinion, this mother accepts individual differences in parenting beliefs and practices. She voluntarily chooses to sleep close to her infant for a security reason.

#### Interrater reliability for parental ethnotheoretical themes

Based on 20% of the data, two coders achieved 80% agreement on selecting ethnotheoretical sentences that represented one of the eight themes. Disagreements were resolved by reaching consensus through discussion. At the next step, 20% of ethnotheoretical sentences were categorized by both coders into one of the eight themes. Inter-rater reliability for categorizing ethnotheries was very good (kappa = 0.85). This reliability between a coder coding from Japanese and a coder coding the same material from English assured the accuracy of the translations of value statements. The Japanese coder coded the remaining 80% of the data from the original Japanese.

#### Coding of ethnotheoretical discourse with infrequent themes

For the 31% of participants who did not express any of the eight identified themes, their cultural values were determined by obtaining group consensus from experts in the theory and classified into one of the three cultural models. Examples of *Gemeinschaft*-adapted, *Gesellschaft*-adapted, and mixed *Gemeinschaft*- and *Gesellschaft*-adapted values obtained by this process are as follows:

**Example of a Gemeinschaft-adapted cultural value**. *… No one around me has their babies sleep alone in a separate room. In my family, I take care of my baby at night, since my husband would never wake up until morning whatever happens. Babies, who cannot turn over yet, are at risk of suffocation and SIDS because they would not be able to remove a blanket by themselves if it covers over their face. In my case, I sleep with my older child and baby. By the way, my husband sleeps in a separate room because of his bad snoring*.

This mother's posting indicates that she sleeps with her baby, perhaps, because of her concerns about the possibility of the baby's “*suffocation and SIDS*.” It is clear, however, that she is a primary caregiver, excluding her husband from sharing sleep environment with their children due to “*his bad snoring*.” This relates to Vogel's report (1963) that the basic alignment in Japanese families is mother and children vs. father, which indicates the strong interdependency of mother and child. Thus, this mother's cultural value was considered *Gemeinschaft*-adapted.

**Example of a Gesellschaft-adapted value**. *I have a six-month-old girl. My friends around me don't sleep with their baby on the same bed or on the same futon. Hmm, I know one mother [who co-sleeps with her baby]. In my family, my husband and I sleep on a bed, and the baby in a crib by the window. I've been doing this since I got out of hospital. I rarely sleep at the same time when the baby sleeps, so I [usually] talk with my husband or do domestic work after baby falls asleep. So I am not always physically close to the baby even though we sleep in the same room*.

This mothers' posting gives the impression that she is somewhat emotionally detached from her baby. The sentences, *“In my family, my husband and I sleep on a bed, and the baby in a crib by the window,”* suggests that she may prioritize intimacy with her husband over the baby. This implies a *Gesellschaft*-adapted value, mother's independence from the child.

**Example of mixed Gemeinschaft- and Gesellschaft-adapted values**. *The definition of “soine” is unclear to me. Does it mean that a mother and a baby sleep together on the same bedding? I think that's scary. In my family, husband and I sleep on our own futon next to each other, and baby's futon was spread above my head. So this is not “kawa”* [child sleeping between parents] *position. But with this position, my husband and I are close to each other, and plus, I can see my baby's face. I wonder whether it's difficult to breastfeed a baby or change diapers when sleeping in a separate room. Just a simple question*.

US parents value a marital bond, while Japanese parents traditionally value a mother-child bond (Befu, 1986). The former may indicate a *Gesellschaft*-adapted value, mother's independence from the child, whereas the latter may represent interdependence between mother and child. The following sentences, *“In my family, husband and I sleep on our own futon next to each other, and baby's futon was spread above my head,”* and *“my husband and I are close to each other, and plus, I can see my baby's face,”* indicate that she values intimacy with her husband, but at the same time, maintains physical, and perhaps emotional, closeness to her baby. Her cultural value was, thus, classified as a mixture of *Gemeinschaft-* and *Gesellschaft-*adapted values.

#### Statistical analysis

Because of having categorical data, we used the binomial test and the Freeman-Halton extension of the Fisher's exact test. The binomial test was used to test whether the uniform *Gesellschaft*-adapted values associated with mother and child sleeping in a separate room could have occurred by chance. The Freeman-Halton extension of the Fisher's exact test was used to test an overall association between the two major categories of sleeping arrangements (same room, different room) and the mothers' value system (*Gemeinschaft*-adapted, *Gesellschaft*-adapted, mixed). Because this analysis involved a 2 × 3 design, the Freeman-Halton extension permitted the Fisher's (a chi-square test for small samples) to be extended from a 2 × 2 design to a 2 × 3 design.

## Results

### Constancy of sleeping arrangements in japan over historical time

Seventy two percentage of our 2008-2009 participants reported sleeping with their babies Within Arms' Reach in the Same Room (*n* = 37). 20% of mothers reported their babies to sleep Beyond Arms' Reach in the Same Room (*n* = 10). Only 8% reported that their babies slept Beyond Arms' Reach in a Separate Room (*n* = 4).

To compare our data with prior decades (Caudill and Plath, 1966; Ohkubo, 2005), we combined Within Arms' Reach in the Same Room (72%) and Beyond Arms' Reach in the Same Room (20%): 92% of Japanese mothers in 2008–2009 slept with their babies in the same room. Thus, the percentage of co-sleeping remained consistently high across the three generational cohorts: 90% in the 1960s (Caudill and Plath, 1966), 99% in the 1980s (Ohkubo, 2005), and 92% in 2008–2009 (this study) for sleeping in the same room; 10, 1, and 8% respectively for baby sleeping alone.

### Relationship between practices and values

Contrary to their sleeping practices, the value statements of our participants were rather equally distributed among different cultural models: 33% of mothers (*n* = 17) expressed only *Gemeinschaft*-adapted values, 39% (*n* = 20) expressed only *Gesellschaft*-adapted values, and 28% (*n* = 14) expressed mixed values. Across the three types of sleeping practices, *Gesellschaft*-adapted values (39%) were more frequent than *Gesellschaft*-adapted practices (8%).

**Figure 1:**
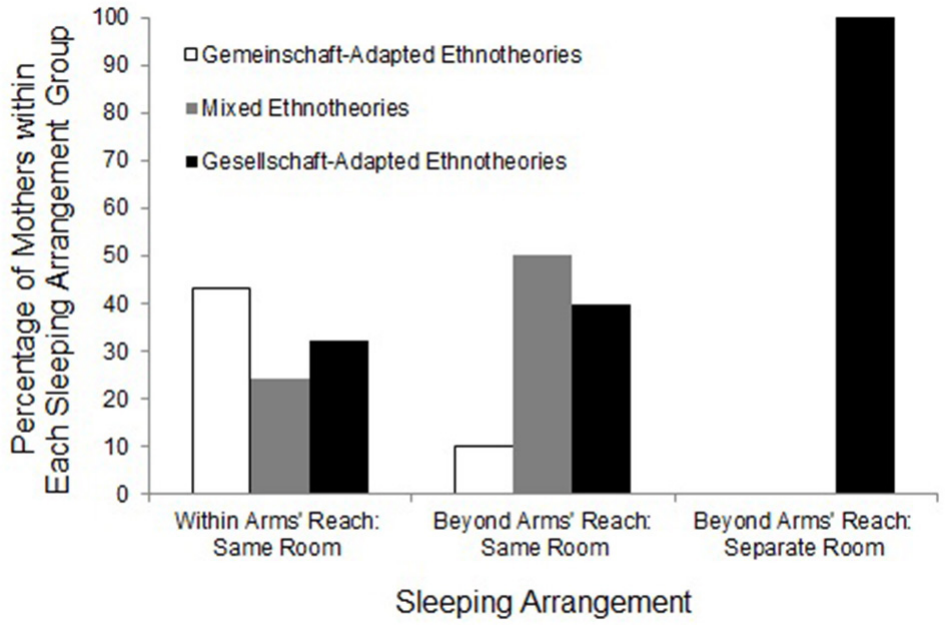
**Relationship between sleeping arrangement practices and maternal ethnotheories**.

Inspecting Figure 1, we see that the highest proportion of mothers expressing *Gemeinschaft*-adapted values had babies who slept within arms' reach. The highest proportion of mothers expressing *Gesellschaft*-adapted values had mothers whose babies slept in a separate room. Moreover, the intermediate sleeping arrangement, Beyond Arms' Reach in the Same Room, was associated with the highest proportion of mothers expressing mixed values. That is, a compromise sleeping arrangement between the two extremes was the one most often supported by mixtures of both *Gemeinschaft*- and *Gesellschaft*-adapted values. This pattern indicated that different infant sleeping practices were associated with different sets of values.

Using the sleeping-arrangement categories of the older Japanese studies, we used a Fisher exact test with Freeman-Halton extension to compare the ethnotheories of mothers who slept in the same room with their babies with those who did not. The ethnotheories of the two groups were significantly different (*p* = 0.033). All four of the mothers who slept in a different room from their baby expressed pure Gesellschaft values; in contrast, only 34% (*n* = 16) of the 47 mothers who slept in the same room with their babies expressed such values.

In terms of variability, Figure 1 shows no variability in the cultural values for the four mothers who reported babies sleeping Beyond Arms' Reach in a Separate Room; all four mothers whose infants slept alone in a separate room expressed *Gesellschaft*-adapted values. Given the possibility in maternal ethnotheories of three different types of value statements (*Gesellschaft* adapted, *Gemeinschaft* adapted, and mixed), the chance probability of any one type of ethnotheoretic statement is 0.33. A binomial test showed that the probability of all four mothers having *Gesellschaft* adapted ethnotheories by chance is 0.012.

Variability in parental ethnotheories among mothers who reported babies sleeping Beyond Arms' Reach in the Same Room showed an expected pattern: the percentage of mixed values was highest (50%, *n* = 5), followed by *Gesellschaft*-adapted values (40%, *n* = 4). The percentage of *Gemeinschaft*-adapted values was lowest (10%, *n* = 1). In other words, the intermediate sleeping arrangement—baby sleeping Beyond Arms' Reach in the Same Room—was most often accompanied by mixed values.

In contrast, variability in parental ethnotheories among mothers who reported babies sleeping Within Arms' Reach in the Same Room was a partly unexpected pattern: as expected, the percentage of *Gemeinschaft*-adapted values was highest (43%, *n* = 16). However, *Gesellschaft*-adapted values (33%, *n* = 12) were the second most frequent ethnotheoretic category—this was not expected. The percentage of mixed values was lowest (24%, *n* = 9). Whereas mothers practicing a *Gesellschaft*-adapted sleeping arrangement showed consistency with their ethnotheories, more than half of the co-sleeping mothers showed at least some discrepancy between their *Gemeinschaft*-adapted practices and parental ethnotheories. These discrepancies were manifest in the discourse of dissatisfaction, the subject of a later section. First we start with some examples from the largest group, mothers who slept within arms' reach of their babies and expressed purely *Gemeinschaft*-adapted values.

### The majority discourse and practice: *gemeinschaft*-adapted values + baby sleeps within arms' reach

Hitomi expresses her ascribed gender role as a mother, as well as her co-sleeping practice:

*I have a 20-day-old first baby. In our family, my baby and I sleep in a tatami room on the first floor, and my husband sleeps on the second floor. My son often wakes up every two hours. He cries at least two times from 12:00 to 7 a.m. … My husband sometimes needs to drive long distances. We [husband and I] decided to sleep in a separate room, since I am afraid that he would have an accident due to sleep deprivation. We need my husband to bring money, so I think of child rearing as a part of my job*.

Arisa expresses the value of security engendered by being close to her child, as well as her practice of sleeping in the same bed with her child:

*I needed to be close to my baby when [he/she] was little because [he/she] frequently threw up milk. I couldn't sleep, worrying if [he/she] would die from clogged milk. I haven't even thought about having [him/her] sleep in a separate room. [He/She] is now 2 years-old, but we sleep together on the same bed because [he/she] vomited twice last year. I will continue [sleeping together with the child] until [he/she] becomes four or five years old*.

### Traditional practice and values with acceptance of individual differences: mixed values + baby sleeps within arms' reach

Many of our participants who sleep with their baby in the same room, in fact, acknowledge individual differences, a *Gesellschaft*-adapted value, by accepting baby's sleeping in a separate room alone as legitimate practice, although they themselves prefer traditional, *Gemeinschaft*-adapted practices and values. For example, Mutsumi responds to Yuki, whose baby sleeps in a separate room:

*I do soine [co-sleeping] with my four-year-old child and one-year-old baby.… I feel peaceful by holding the baby's hand, seeing [his/her] face, and listening to [his/her] breathing* [while we sleep together]. *[But,] you and your husband together decided to have your baby sleep in a separate room alone; I think it is acceptable*.

Mutsumi feels happiness from physical contact and proximity, which represents a *Gemeinschaft-*adapted value. However, she also values the importance of personal choice, a *Gesellschaft-*adapted value.

### The discourse of dissatisfaction: *gesellschaft*-adapted values + baby sleeps within arms' reach

Qualitative analysis of discourse revealed the subjective aspect of social change. The discrepancy between *Gemeinschaft*-adapted practice and *Gesellschaft*-adapted ethnotheory was quite common: As noted earlier, 12 of the 37 mothers who slept with their babies within arms' reach expressed *Gesellschaft*-adapted values. This quantitative fact is illustrated by qualitative comments from two mothers; both demonstrate dissatisfaction with the traditional gender role regarding childrearing, which places the responsibility solely on mothers. Reiko expresses a wish for gender egalitarianism in the parenting role:

*I have a two-year-old daughter, and am now pregnant. [We: baby, mother, and father] sleep together in a tatami room in the ‘kawa’ [child sleeping between parents] position. My husband doesn't wake up [to help me]. But, I think it is important to let [husbands] notice the baby's crying. I guess it is better to let [husbands] know on a daily basis that child-rearing is hard work than to let the busy husbands participate in child rearing only on weekends*.

On a different forum, Izumi commented:

Males always say, ‘I would be sleep deprived by the baby's night crying.’ I was upset, thinking why he said that as if he were a third person. This is our baby. So, I let him [forced him to] sleep together with us. Even though the baby cried, he has never awakened. I immediately wake up”

Sleeping arrangements for these women are not about the baby and her psychological development, but about adult gender roles.

### A compromise between tradition and change: mixed values + baby sleeps beyond arms' reach in same room

Mika also responds to Yuki, the mother whose baby sleeps in a separate room:

I have a three-month-old daughter. She sleeps in a crib in my bedroom. I will give her a room when she says that she wants to have it. As many mothers say, I think it is acceptable to have a baby sleep in a separate room alone. Mother's love never fades away [even though she lets her baby sleep in a separate room]. I have my baby sleep next to me [my bed] just because I can't sleep, worrying too much [about her].…

Here Mika emphasizes the importance of personal choice, an individualistic *Gesellschaft*-adapted value; yet she chose to sleep with her baby in the same room because being close to her baby makes her feel secure. Her mixed values mirror her intermediate sleeping arrangement.

### Grappling with new societal conditions: *gesellschaft*-adapted values + baby sleeps in a separate room or beyond arms' reach in the same room

Mothers with babies and toddlers sleeping in a room alone often made it clear that they were adapting to new economic realities. Here is one such posting by Yuki:

*I leave home by 9:30 a.m., so I think I want to keep at least a 3-hour-sleeping time for myself. […] Some people often told me, ‘Stop working for a while. You have to rear your child,’ but both [child-rearing and work] are important to me, I can't quit my job*.

This post expresses a conflict between societal expectations and the personal value placed on independence. However, Yuki finds a way to care for her baby while maximizing the independence of both: “I wake up and go to my baby's room to give him milk every three hours by timing with a timer.”

This use of the timer also illustrates that technology is an important characteristic of a *Gesellschaft* environment. Valuing technology is of course adaptive in such an environment. In this group, we saw the high technology commonly available in today's Japan as another factor in letting babies sleep alone in their own room. Kumi responds to Yuki:

*If you don't feel secure, you can put a camera and microphone in the corner of the crib. Cameras and microphones are well developed, so you can check baby's breathing from your room*.

The following quote from Nanami, also in response to Yuki, manifests how some Japanese mothers are developing new values in response to current economic realities:

*I started to work a week after my baby was born. … My husband also has a job, but he and I sleep with [our] baby in the same room. … My husband wanted to have a child, but I didn't want to, and I didn't want to do child-rearing or sleep alongside a baby [soine]. … As a result, [we] put a crib next to our bed in our room so that I can wake up by hearing even a little noise…. It is the responsibility of a husband and a wife that you both work and have a child*.

This quote suggests that she does not want to follow the traditional practice of co-sleeping and perceives childrearing as a cooperative job between husband and wife; it exemplifies another *Gesellschaft*-adapted value, egalitarian gender roles and movement away from traditional Japanese hierarchical gender roles (Yashiro, 2001).

## Discussion

The current study examined parenting practices and underlying cultural values of Japanese mothers in 2008-2009. We predicted a decline in co-sleeping practices in 2008-2009, compared with the 1960s and 1980s, along with consonant child-rearing goals and values. Contrary to our hypothesis, co-sleeping was as frequent among Japanese mothers in 2008-2009 as it had been in the 1960s and 1980s. However, analysis of the parental ethnotheories revealed frequent discrepancies between ethnotheories and practices among mothers who co-slept with their babies.

Stability in sleeping arrangements across the three generational cohorts suggests that co-sleeping is indeed the common childrearing practice even in contemporary Japan. This is similar to the finding that emerged from a large national Internet survey utilizing data collected in 2007 (Kohyama et al., 2011). One possible explanation for the lack of changed practices is the space limitation in Japan, as sleeping arrangements are a function of both preferences and constraints (Shweder et al., 1995). Despite the rapid social change, space in the home is limited in contemporary Japan and often does not allow children to have their own rooms. However, our findings do not seem to support this conclusion as the overwhelming majority (79%) of those sleeping in the same room slept within arms' reach of their babies. If parents indeed preferred distal sleeping arrangement, but simply could not sleep in a separate room due to limited number of rooms in the home, we would expect parents to maximize parent-child distance within the same room (Beyond Arms' Reach in the Same Room).

Although we found that in contemporary *Gesellschaft* Japan the majority of mothers co-slept with their babies—a practice that is normative in a *Gemeinschaft* environment and is the traditional Japanese practice—we also found that mothers who practiced co-sleeping frequently expressed conflicts. These expressed conflicts illuminated the dynamics of coping with social change, such as maternal employment, that make traditional co-sleeping practices less adaptive. Although it is theoretically possible to combine co-sleeping with gender equality vis-à-vis infant care (and this probably happens in the US, which has continued to move in the *Gesellschaft* direction even during the 21st century, Greenfield, 2013), there was no sign of father participation in infant care during co-sleeping in our Japanese protocols.

On the other hand, mothers whose babies slept alone in a separate room invariably held consonant *Gesellschaft*-adapted values without expressions of conflict. That is, mothers practicing *Gesellschaft*-adapted distal sleeping arrangement showed consistency with their values, whereas most of co-sleeping mothers showed some discrepancy between their *Gemeinschaft*-adapted co-sleeping practices and values. The findings should be interpreted cautiously given the small proportion of mothers who slept with their babies in a separate room (8%), but co-sleeping is indeed the historically normative arrangement. Co-sleeping has been supported by traditional ethnotheories that value physical proximity and foster the child's interdependent functioning (Fogel et al., 1992). While cultural values and infant sleeping arrangements have not been assessed in the same study, separate studies have indicated that, in past decades, other *Gemeinschaft*-adapted values, notably maternity as an ascribed role and gender hierarchy, were important in Japanese culture (Hirao, 2001; Greenfield, 2009). Therefore, our study provides a preliminary conclusion that ethnotheories may be changing in advance of practices. Future research should continue to follow sleeping arrangements in Japan in order to find out whether and when changes in the practice take place.

One possible reason why Japanese mothers practice co-sleeping despite having dissonant independence-oriented cultural values may be due to the societal expectation that remains in contemporary Japan. For instance, despite mothers' movement toward gender egalitarianism, mothers in our study reported that they had expectations about childrearing roles that conflicted with those of their husbands. Mothers also described societal expectations, such as quitting their jobs to raise children, even though they themselves did not desire it. Thus, future research should take a closer look at mothers' internal conflicts arising from changing parental ethnotheories in the midst of resistant cultural expectations, and their implications for family relationships and well-being.

Utilizing Internet bulletin boards, our study captured candid opinions of Japanese mothers who could experience discomfort expressing true emotions in research contexts (Rice, 2001). Their open discussion about disagreement with societal expectations corroborates this claim that Internet bulletin boards provided a good platform for gathering parental ethnotheories and gender-role values in Japan. Indeed, the anonymous nature of Internet communication uncovered tensions created by traditional infant care practices and gender roles in a new sociodemographic environment. Furthermore, Internet boards allow mothers' opinions to be communicated to a potentially mass audience, constituting cultural products that go beyond the significance for the individuals posting their views.

Given the nature of Internet bulletin boards, we could not obtain sample details such as specific region of residence in Japan. Thus, our study was not able to assess selection bias of the sample or the representativeness of our sample for Japan as a whole. Therefore, it is possible that mothers with more conflicts about childrearing may be more drawn to Internet discussion boards. However, the forum titles were general and did not include words such as conflict or trouble with parenting or co-sleeping. In addition, high Internet penetration in Japan at the time of data collection (75%, Internet World Stats, 2009) reduced the selectivity of our sample.

This was a mixed-method study, combining quantitative and qualitative analysis. On the qualitative level, the conversational quality of the bulletin boards—mothers responding to each other—combined with the anonymity of participants' discourse to strengthen self-expression.

On the quantitative level, the conversational nature of Internet bulletin boards meant that participants' data were not always independent of one another, therefore violating an underlying assumption of inferential statistics. However, this factor would not affect our particular statistical analyses as these analyses assessed within-subject relations (between ethnotheory and practice) rather than between-subject differences; the latter, but not the former, could have been compromised by interdependence between conversational turns. In addition, some mothers initiated expression of their own parental ethnotheories and their sleeping arrangements, rather than responding to previous comments. Even more significant, mothers' own parental ethnotheories and sleeping arrangements often were not in agreement with those of mothers who had posted their comments earlier. This fact suggests that our participants were candidly expressing their views, rather than merely agreeing with the views of others.

Although the anonymity of Internet forums prevented us from obtaining personal information about the mothers, it provided major advantages concerning mothers' disclosure of values and possible conflicts regarding childrearing and sleeping arrangements. Parenting bulletin boards on the Internet provided a supportive group atmosphere plus anonymity; these features allowed participants to express feelings that would not have been considered acceptable according to traditional Japanese norms. These bulletin boards therefore provide a unique window into Japanese mothers' values and inner conflicts.

## Author notes

The authors would like to thank the Greenfield Laboratory group for their feedback on the paper and assistance in coding the ethnotheoretical discourse with infrequent themes. Author Park is now at Beloit College in Wisconsin.

### Conflict of interest statement

The authors declare that the research was conducted in the absence of any commercial or financial relationships that could be construed as a potential conflict of interest.

## References

[B1] Abbott S. (1992). Holding on and pushing away: comparative perspectives on an Eastern Kentucky child rearing practice. Ethos 20, 33–65 10.1525/eth.1992.20.1.02a00020

[B2] Azuma H. Kashiwagi K. Hess R. (1981). The Influence of Attitude and Behavior upon the Child's Intellectual Development. Tokyo: University of Tokyo Press

[B3] Befu H. (1986). The social and cultural background of child development in Japan and the United States, in Child Development and Education in Japan, eds Stevenson H. Azuma H. Hakuta K. (New York, NY: W. H. Freeman), 13–27

[B4] Brazelton T. B. (1990). Commentary: parent-infant cosleeping revisited. Ab Initio 2, 1–7

[B5] Brazelton T. B. Robey J. S. Collier G. A. (1969). Infant development in the Zinacanteco Indians of Southern Mexico. Pediatrics 44, 274–290 5806260

[B6] Caudill W. Plath D. W. (1966). Who sleeps by whom?: parent-child involvement in urban Japanese families. Psychiatry 29, 344–366 10.1080/00332747.1966.11023478 27820920

[B7] Caudill W. Weinstein H. (1969). Maternal care and infant behavior in Japan and America. Psychiatry 32, 12–34 5779087 10.1080/00332747.1969.11023572

[B8] DePaulo B. (2006). Singled Out: How Singles are Stereotyped, Stigmatized, and Ignored, and Still Live Happily Ever After. New York, NY: St. Martin's Press

[B9] Efron S. (2001, June 24–26). Japan's demography shock. Los Angeles Times, pp. A1, A9 12285109

[B43a] Fogel A. Stevenson M. B. Messinger D. (1992). Parenthood in Japan and the United States: Its relationship to early childhood development, in Parent-Child Relations in Diverse Cultural Settings, eds Roopnarine J. L. Carter D. B. (Norwood, NJ: Ablex), 35–51

[B10] Greenfield P. M. (2009). Linking social change and developmental change: shifting pathways of human development. Dev. Psychol. 45, 401–418 10.1037/a0014726 19271827

[B43b] Greenfield P. M. (2013). The changing psychology of culture from 1800 through 2000. Psychol. Sci. 24, 1722–1731 10.1177/0956797613479387 23925305

[B11] Greenfield P. M. Keller H. Fuligni A. Maynard A. (2003). Cultural pathways through universal development. Annu. Rev. Psychol. 54, 461–490 10.1146/annurev.psych.54.101601.145221 12415076

[B12] Greenfield P. M. Suzuki L. K. (1998). Culture and human development: implications for parenting, education, pediatrics, and mental health, in Handbook of Child Psychology: Vol. 4. Child Psychology in Practice, 4th Edn., eds Sigel I. E. Renninger K. A. (New York, NY: Wiley), 1059–1109

[B13] Harkness S. Super C. S. Axia V. Eliasz A. Palacios J. Welles-Nystrom B. (2001). Cultural pathways to successful parenting. ISSSBD Newsletter, no. 1, Serial no. 38, 9–13

[B14] Hirao K. (2001). Mothers as the best teachers: Japanese motherhood and early childhood education, in Women's Working Lives in East Asia, ed Brinton M. C. (Stanford, CA: Stanford University Press), 180–203

[B15] Hofstede G. H. (1980). Culture's Consequences: International Differences in Work-Related Values (Abridged ed.). Beverly Hills, CA: Sage

[B16] Internet World Stats. (2009). Usage and Population Statistics. Available online at: http://www.internetworldstats.com/asia/jp.htm

[B17] Kağitçbaşl Ç. (2005). Autonomy and relatedness in cultural context: implication for self and family. J. Cross Cult. Psychol. 36, 403–422 10.1177/0022022105275959

[B18] Keller H. (2007). Cultures of Infancy. Mahwah, NJ: Lawrence Erlbaum Associates

[B19] Kohyama J. Mindell J. Sadeh A. (2011). Sleep characteristics of young children in Japan: Internet study and comparison with other Asian countries. Pediatr. Int. 53, 649–655 10.1111/j.1442-200X.2010.03318.x 21199167

[B20] Lebra T. S. (1976). Japanese Patterns of Behavior. Honolulu, HI: University of Hawaii Press

[B21] Lebra T. S. (1994). Mother and child in Japanese socialization: a Japan-U.S. comparison, in Cross-Cultural Roots of Minority Child Development, eds Greenfield P. M. Cocking R. R. (Hillsdale, NJ: Erlbaum), 259–273

[B22] Li J. (2012). Cultural Foundations of Learning. New York, NY: Cambridge University Press

[B23] Manago A. M. Greenfield P. M. (2011). The construction of independent values among maya women at the forefront of social change: four case studies. Ethos 39, 1–29 10.1111/j.1548-1352.2010.01168.x

[B24] Manago A. M. Greenfield P. M. Kim J. L. Ward L. M. (2014). Changing cultural pathways through gender role and sexual development: a theoretical framework. Ethos 42, 198–221 10.1111/etho.12048

[B25] Markus H. R. Kitayama S. (1991). Culture and the self: Implications for cognition, emotion, and motivation. Psychol. Rev. 98, 224–253 10.1037/0033-295X.98.2.224

[B26] McKenna J. J. McDade T. (2005). Why babies should never sleep alone: a review of the cosleeping controversy in relation to SIDS, bedsharing and breast feeding. Pediatr. Resp. Rev. 6, 134–152 10.1016/j.prrv.2005.03.006 15911459

[B27] Ministry of Education, Culture, Sports, Science, and Technology. (2009). Heisei 21nenndo Gakkoukihonnchousasokuhou Chousakekkanoyoushi. [Heisei21Preliminary Research Findings Summary of the Basic School Year]. Available online at: http://www.mext.go.jp/b_menu/toukei/001/08121201/1282588.htm

[B28] Morelli G. A. Rogoff B. Oppenheim D. Goldsmith D. (1992). Cultural variation in infants' sleeping arrangements: questions of independence. Dev. Psychol. 28, 604–613 10.1037/0012-1649.28.4.604

[B29] Niimura I. (ed.). (2008). Kojienn, 6th Edn. Tokyo: Iwanami

[B30] Ohkubo T. (2005). Two types of cosleeping: child centered type and mother centered type, in NFRJ-S01 Report No. 2: Trails of Families in Post-War Japan, eds Kumagai S. Ohkubo T. (Tokyo: Japan Society of Family Sociology, NFRJ Committee) 113–126

[B31] Pruett K. (1987). The Nurturing Father. New York, NY: Warner Books

[B32] Rice Y. N. (2001). The maternal role in Japan: cultural values and socioeconomic conditions, in Japanese Frames of Mind: Cultural Perspectives on Human Development, eds Shimizu H. LeVine R. A. (New York, NY: Cambridge University Press), 85–110

[B33] Rosenberger N. (2001). Gambling with Virtue: Japanese Women and the Search for Self in a Changing Nation. Honolulu, HI: University of Hawaii Press

[B34] Shweder R. A. Jensen L. A. Goldstein W. M. (1995). Who sleeps by whom revisited: A method for extracting the moral goods implicit in practice. New Dir. Child Adolesc. Dev. 1995, 21–39 10.1002/cd.23219956705 7566543

[B35] Super C. M. Harkness S. (1986). The developmental niche: a conceptualization at the interface of child and culture. Int. J. Behav. Dev. 9, 545–569 10.1177/016502548600900409

[B36] Takahashi K. (1990). Are the key assumptions of the “strange situation” procedure universal?: a view from Japanese research. Hum. Dev. 33, 23–30 10.1159/000276500

[B37] Tönnies F. (1887/1957). Community and Society (Gemeinschaft und Gesellschaft), ed Trans Loomis. C. P. East Lansing, MI: Michigan State University Press (Original work published in German, 1887).

[B38] Vogel E. F. (1963). Japan's New Middle Class: the Salary Man and his Family in a Tokyo Suburb. Berkeley, CA: University of California Press

[B39] Weisner T. S. Bausano M. Kornfein M. (1983). Putting family ideals into practice. Ethos 11, 278–304 10.1525/eth.1983.11.4.02a00060

[B40] Welles-Nystrom B. (2005). Cosleeping as a window into Swedish culture: considerations of gender and health care. Scand. J. Caring Sci. 19, 354–360 10.1111/j.1471-6712.2005.00358.x 16324059

[B41] Wolf A. W. Lozoff B. Latz S. Paludetto R. (1996). Parental theories in the management of young children's sleep, in Parents' Cultural Belief Systems: their Origins, Expressions, and Consequences, eds Harkness S. Super C. M. (New York, NY: Guilford Press), 364–384

[B42] World Bank. (2010). World Development Indicators (WDI) & Global Development Finance (GDF). Available online at: http://databank.worldbank.org/ddp/home.do?Step=3&id=4

[B43] Yashiro N. (2001). Social implications of demographic change in Japan, in Seismic Shifts: the Economic Impact of Demographic Change, eds Little J. S. Triest R. K. (Boston: Federal Reserve Bank of Boston), 297–304

