# Supplementary material for: Infant sleeping arrangements and cultural values among contemporary Japanese mothers
Source: Front Psychol. 2014 Aug 19;5:718. doi: 10.3389/fpsyg.2014.00718 (PMC4137277; doi:10.3389/fpsyg.2014.00718)
Supplement: Supplementary file 1 [file Appendix_Japanese_Online_Parenting_Forums.DOCX]

Appendix

Data in this study came from the following 15 Japanese online parenting forums.

*Akachannbeya no tsukurikata, adobaisukudasai* [Please give me some advice on where a baby should sleep]. Retrieved April 16, 2009, from <http://komachi.yomiuri.co.jp/t/2008/1217/217160.htm?g=05>.

*Akachann no bed to fuufu no bed ha issyonoheya??* [Are a crib and a parental bed in the same room??].^1^ Retrieved April 17, 2009, from http://www.qa.findstar.co.jp/qa4853559.html.

*Akachann no nerubasyo, baby bed wo okubasyonitsuite* [The place where a baby sleeps, the place where a crib is placed]. Retrieved March 1, 2009, from http;//oshiete1.goo.ne.jp/qa4179283.html.

*Akachann no shinnshitsu* [The place where a baby sleeps]. Retrieved April 17, 2009, from <http://onayamifree.com/dispthrep.cgi?th=705256&allres=1>.

*Baby bed* [Cribs].^2^ Retrieved March 1, 2009, from <http://scomu.jp/bedfbaby/>.

*Baby bed, futon, kuhfann nadono shinnguyouhinn* [Bedding such as cribs, *futons*, *kuhfanns*]. Retrieved April 12, 2009, from <http://www.pixy.cx/~kamosika/cbbs/cbbs.cgi?mode=al2&namber=1&page=0&rev=1&no=0>.

*Baby bed ka ofuton kade nayanndeimasu* [I am wondering which is better, a crib or a futon]. Retrieved April 12, 2009, from <http://oshiete1.watch.impress.co.jp/qa4042375.html>.

*Baby bed ha hitsuyoudeshitaka?* [Did you use a crib?].^3^ Retrieved April 16, 2009, from <http://kaiketsu.athome.jp/qa4820628.html>.

*Baby bed nitsuite* [Regarding cribs].^4^ Retrieved April 17, 2009, from <http://com.babycome.ne.jp/U003.php?article_id=118381>.

*Baby bed nitsuite soudann* [A question regarding cribs]. Retrieve April 17, 2009, from <http://okwave.jp/qa4867383.html>.

*Beby bed to ofuton* [Cribs and *futons*]. Retrieved April 12, 2009, from <http://okwave.jp/qa3994045.html>.

*Kodomogaumaretara, Bed?Futon??* [Which one did you use when you gave birth to your baby, a crib? or a *futon*??]. Retrieved April 17, 2009, from <http://questionbox.jp.msn.com/qa3664144.html>.

*Kodomowonekaserubasyo ha doushiteimasuka?* [Where does your baby sleep?]. Retrieved April 12, 2009, from <http://detail.chiebukuro.yahoo.co.jp/qa/question_detail/q1414520509?fr=rcmd_chie_detail>.

*Nerukannkyoudesuga…* [Regarding sleeping environment…].^5^ Retrieved April 16, 2009, from <http://com.babycome.ne.jp/U003.php?article_id=119732>.

*Umareteikkagetsudebetsunoheyaninekaseteimasu* [My one-month-old baby sleeps in a separate room]. Retrieved April 17, 2009, from http://komachi.yomiuri.co.jp/t/2008/1007/207245.htm?o=0&p=0.

Note

^1, 2, & 3^ These websites are no longer operative.

^4 & 5^ These threads were deleted, and are no longer available.
